# How do we prevent severe intra‐abdominal infectious complications following minimally invasive gastrectomy for cancer? The usefulness of a novel marker using computed tomography images (minimum umbilicus–vertebra diameter) and robotic surgery

**DOI:** 10.1002/ags3.12760

**Published:** 2023-12-03

**Authors:** Naoshi Kubo, Katsunobu Sakurai, Tsuyoshi Hasegawa, Junya Nishimura, Yasuhito Iseki, Takafumi Nishii, Sadatoshi Shimizu, Toru Inoue, Yukio Nishiguchi, Kiyoshi Maeda

**Affiliations:** ^1^ Department of Gastroenterological surgery Osaka City General Hospital Osaka Japan; ^2^ Department of Gastroenterological surgery Osaka Metropolitan University Graduate School of Medicine Osaka Japan

**Keywords:** CT image, gastric cancer, intra‐abdominal infectious complications, robotic gastrectomy

## Abstract

**Background:**

Intra‐abdominal infectious complications (IAICs) following minimally invasive gastrectomy (MIG) for cancer sometimes worsen short‐ and long‐term outcomes. In this study, we focused on the minimum umbilicus–vertebra diameter (MUVD) in preoperative computed tomography (CT) images and robotic surgery to prevent severe IAIC occurrence.

**Patients and Methods:**

A total of 400 patients with gastric cancer who underwent 204 laparoscopic gastrectomy (LG) and 196 robotic gastrectomy (RG) procedures were enrolled in this study. We retrospectively investigated the significance of the MUVD and robotic surgery for preventing severe IAICs following MIG using multivariate and propensity score matching analysis.

**Results:**

The MUVD cutoff value was 84 mm by receiver operating characteristic (ROC) curve using severe IAICs as the end point. The MUVD and visceral fat area (VFA) had significantly higher area under the curve (AUC) than BMI (MUVD vs. BMI, *p* = 0.032; VFA vs. BMI, *p* < 0.01). In the multivariate analysis, high MUVD (HR, 9.46; *p* = 0.026) and laparoscopic surgery (HR, 3.35; *p* = 0.042) were independent risk factors for severe IAIC occurrence. In the propensity matching analysis between robotic and laparoscopic surgery in the high MUVD group, the RG group tended to have a lower severe IAIC rate than the LG group (0% vs. 9.8%, *p* = 0.056).

**Conclusion:**

The MUVD was a novel and easy‐measuring predictor of severe IAICs following MIG. Robotic surgery should be considered first in patients with gastric cancer having an MUVD value of 84 mm or higher from the perspective of severe IAIC occurrence.

## Graphical abstract

The minimum distance between the deepest part of the umbilicus and the vertebral body measured from axial abdominal CT images at the level of the umbilicus is a significant predictor of the occurrence of intra‐abdominal infectious complications after minimally invasive gastric cancer surgery.

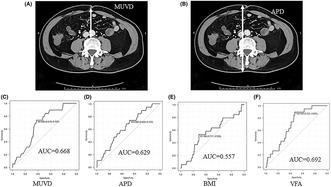

## 1 INTRODUCTION

Among various cancers worldwide, gastric cancer ranks fifth and third in incidence and mortality rates, respectively.^1^ The mainstay for localized gastric cancer is surgical treatment. Recently, minimally invasive gastrectomies (MIGs) including laparoscopic gastrectomy (LG) and robotic gastrectomy (RG) have been focused as more useful surgical approaches than open surgery. However, among the complications following MIG, intra‐abdominal infectious complications (IAICs) including pancreatic fistula, anastomotic leakage, and intra‐abdominal abscess are the main causes of increased length of hospital stay and perioperative mortality. Additionally, it has been reported that IAICs are associated worse long‐term prognosis.^2^, ^3^, ^4^ Therefore, prediction and countermeasures for IAICs following MIG are significant. Various risk factors for IAIC development following gastric cancer surgery have been previously reported, including male sex,^5^ obesity,^6^, ^7^ pancreatic thickness,^8^ laparoscopic surgery compared with open surgery^9^ or robotic surgery,^10^ and total gastrectomy compared with distal gastrectomy.^7^ Among these predictive factors, obesity is one of most significant factors for postoperative IAIC occurrence as the number of patients with obesity has been increasing globally in recent years,^11^ and it is expected that there will be more opportunities to perform surgery on patients with obesity with gastric cancer in Japan in the near future. It has been previously reported that some obesity‐related body parameters measured using preoperative computed tomography (CT) images such as anterior–posterior diameter (APD) were simple and easy‐measuring indicators for IAICs following surgery for patients with gastric cancer.^12^, ^13^ However, no established consensus on its usefulness in predicting IAICs has been observed. Therefore, in this study, we focused on the novel and original obesity‐related parameter, which is the minimum umbilicus–vertebra diameter (MUVD) measured using axial CT image. We hypothesized that the high MUVD is closely associated with severe IAIC occurrence following MIG. This study aimed to clarify the impact of the MUVD on severe IAIC occurrence and compare the MUVD with previously reported obesity‐related indexes, such as BMI, visceral fat area (VFA), and APD, regarding severe IAIC occurrence following MIG. Additionally, we evaluated the usefulness of robotic system in terms of preventing severe IAICs following MIG for patients with high MUVD.

## 2 PATIENTS AND METHODS

### 2.1 Patient selection

Inclusion criteria were as follows: histologically confirmed gastric carcinoma (cT1‐4a, cN0‐3) and minimally invasive gastrectomy associated with radical lymphadenectomy, including RG and LG. A total of 574 patients with local gastric cancer (GC) were surgically treated with curative intent at the Department of Gastroenterological Surgery of Osaka City General Hospital from January 2015 to August 2022. Patients with suspected T4 tumors or bulky metastatic lymph nodes who underwent open surgery (*n* = 122), or esophagogastric junction cancer, including Siewert type I and type II (*n* = 8), or remnant GC (*n* = 7), or GC with another synchronous cancer (*n* = 5) were excluded from this study. In our surgical strategy for localized GC, open surgery was indicated for patients with suspected T4 tumor or bulky metastatic lymph nodes, and minimally invasive surgery (MIS), such as LG or RG, was introduced for patients without highly advanced GC. Furthermore, this study focused on postoperative complications, which are important to maintain the technical safety and quality of surgery; therefore, the surgeons in this study were an experienced four surgeons who held both a technical certification from the Japan Society of Endoscopic Surgery and a da Vinci robotic surgery certificate. Furthermore, the surgeon performed more than 50 laparoscopic surgeries, and met certain technical standards. A total of 32 cases operated by junior surgeons were excluded from this study. Ultimately, 400 gastric cancer patients were enrolled in this study. The pathological diagnosis and classifications of gastric carcinoma were made in accordance with the JGCA guidelines and the Union for International Cancer Control TNM Classification of Malignant Tumors (8^th^ edition).^14^ All data were extracted from a database where patients were prospectively registered. This study was approved by the Ethics Committee of Osaka City General Hospital (No. 1806031). All patients provided their informed consent.

### 2.2 Surgical procedure and postoperative treatment

Surgery was performed with robotic assistance or laparoscopically. Regarding robotic surgery, da Vinci Si or Xi (Intuitive Surgical, Inc.,) was used, and the energy device was mainly an ultrasonic coagulation and cutting device reported by Hyung et al.^15^ or a bipolar device reported by Uyama et al.^16^ The details of the surgery are described in the previous paper.^17^ In addition, lymph node dissection was performed in accordance with the Japanese Gastric Cancer Society guidelines, with D1+ lymph node dissection for early cancer and D2 lymph node dissection for advanced cancer.^18^ After gastrectomy, B‐I or B‐II or Roux‐en‐Y reconstruction was performed, and a drainage tube was inserted into the abdominal cavity. After surgery, patients were treated according to the clinical pathways, and were allowed to start drinking fluids from the next day after surgery and start a soft diet from the third day. Antibiotics were used only during surgery. When postoperative complications occurred, their severity was determined and appropriate interventions (i.e., radiographic or surgical interventions, and conservative pharmacological treatment) were planned. The drain was removed, and patients were able to take sufficient oral intake and to carry out daily activities without any problems. It was determined whether patients were allowed to leave the hospital.

### 2.3 Short‐term surgical outcomes

Postoperative complications were classified according to the Clavien–Dindo (CD) classification.^19^ CD class II or higher was defined as overall complication, and CD class III or higher was defined as a severe complication. In addition, anastomotic leakage, intra‐abdominal abscess, and pancreatic fistulas were defined as intra‐abdominal infectious complications (IAICs). The following parameters were assessed: surgical time, estimated surgical blood loss, number of retrieved lymph nodes. The short‐term outcomes compared between the two groups included surgical time, estimated blood loss, postoperative overall, severe complication rates, details of postoperative complication, and duration of hospital stay.

### 2.4 Body parameters using CT imaging

**FIGURE 1:**
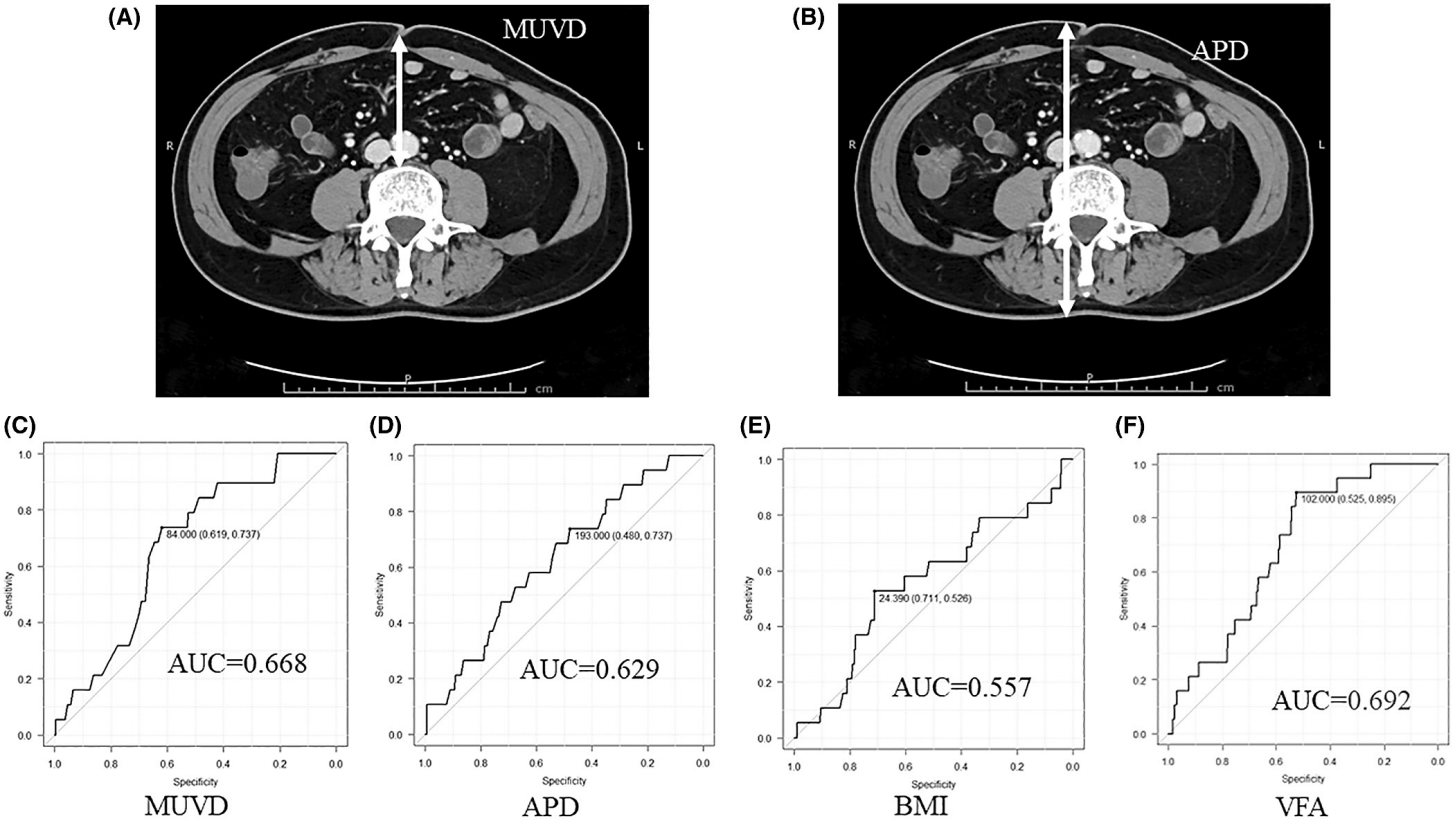
Schema of body parameters using axial computed tomography (CT) images (A,B) and ROC curves of body parameters using the presence or absence of severe IAICs as the end point (C–F). Minimum umbilicus–vertebra diameter (MUVD) is defined as the minimum distance from the deepest point of the umbilicus to the vertebra (Figure 1A). APD is defined as the distance of the anterior abdominal skin to the backside skin at the umbilical level (Figure 1B). The ROC curves of MUVD, APD, BMI, and VFA are shown in Figure 1C–F, respectively. The AUCs of the ROC curves regarding the MUVD and APD are 0.668 and 0.629, respectively; the AUC of the MUVD tends to be higher than that of the APD (*p* = 0.097). The AUCs of the ROC curves regarding BMI and VFA are 0.557 and 0.692, respectively. The DeLong test shows that the MUVD and VFA have significantly higher AUCs than BMI (MUVD vs. BMI, *p* = 0.032; VFA vs. BMI, *p* < 0.01), whereas APD is similar to BMI (APD vs. BMI, *p* = 0.104). Furthermore, the AUCs of the MUVD and VFA are similar (*p* = 0.268). The MUVD cutoff value is determined to be 84 mm from the ROC curve. Sensitivity and specificity for predicting severe IAIC occurrence are 61.9% and 71.7%, respectively.

The following distances were measured in the axial section at the umbilical level in the preoperative CT images to be obtained within 1 month preoperatively (Figure 1A,B). MUVD was defined as the minimum distance from the deepest point of the umbilicus to the vertebra (Figure 1A). APD was defined as the distance of the anterior abdominal skin to the backside skin at the umbilical level (Figure 1B). Moreover, VFA was calculated from axial CT images at the umbilical level.^12^ It was calculated by setting the attenuation level within the range of −200 and −50 threshold units using the image analyzing system SYNAPSE VINCENT® (FUJI Film Corporation) reported in a previous study.^20^

### 2.5 ROC analysis and determination of the best marker for severe IAIC occurrence

ROC curves for the above measured parameters were constructed using the presence or absence of severe IAICs as the end point, and AUC values were measured. The Youden index of each parameter was also calculated from each ROC curve and used as each cutoff point. Among these two parameters, including the MUVD and APD, the most useful marker for IAIC occurrence was determined by the DeLong test using the AUC value of ROC curves,^21^ and all patients were divided into two groups on the basis of the cutoff value of the best marker; subsequently, patient characteristics and short‐term postoperative results were compared between the high and low groups. Furthermore, the ROC curves of the best marker, BMI, and VFA were compared for accuracy as a test for IAIC occurrence using the DeLong test. To investigate the association between the best marker and VFA or BMI, the Pearson correlation analysis was introduced. If the Pearson correlation coefficient (*r*) value was 0.7 or more, two parameters were determined as very high correlation, whereas if the *r* value was between 0.5 and 0.7, two parameters were determined as high correlation. If the *r* value was between 0.3 and 0.5, two parameters were determined as medium correlation, whereas if the *r* value was between 0.3 and 0.1, or 0.1 and 0, two parameters were determined as low or no correlation, respectively.

### 2.6 Multivariate logistic regression analyses of risk factors for severe IAICs

Multivariate analyses of risk factors for severe IAICs were performed using logistic regression analyses. The cutoff values of each examined factor were determined using the Youden index from ROC curves.

### 2.7 Propensity score (PS) matching analysis between the RG and LG groups in terms of short‐term outcomes following MIG for patients with high MUVD

To reduce the heterogeneity of patients' backgrounds in patients with high MUVD, we performed 1:1 PS matching between the RG and LG groups. The PS was calculated as the conditional probability of receiving cases from either group using a logistic regression model and included age, sex, ASA‐PS score, clinical and pathological oncological stage, BMI, VFA, MUVD, gastrectomy type, and the extent of lymph node dissection. Subsequently, we compared the short‐term surgical results, including severe IAICs following surgery between the RG and LG groups.

### 2.8 Statistical analysis

Continuous variables were compared using Mann–Whitney's *U* test, and categorized variables were compared using the chi‐squared test or the Fisher's exact test. A *p* value of <0.05 or less was considered significant. SPSS software and EZR software were used for data analysis.

## 3 RESULTS

### 3.1 ROC curves of each body parameter

Figure 1C–F shows the ROC curves for MUVD, APD, BMI, and VFA, respectively. The AUC areas of MUVD and APD were 0.668 and 0.629, respectively. The AUC of ROC curve for MUVD tend to be higher than that of APD (*p* = 0.092). The AUC of BMI and VFA were 0.557 and 0.692, respectively, and the DeLong test showed that MUVD and VFA had significantly higher AUC than BMI (MUVD vs. BMI, *p* = 0.032; VFA vs. BMI, *p* < 0.01), whereas APD was similar to BMI (MAPD vs. BMI, *p* = 0.104). Furthermore, the AUCs of MUVD and VFA were similar (*p* = 0.268). The MUVD cutoff value was determined to be 84 mm from the ROC curve. Sensitivity and specificity for predicting IAICs occurrence was 61.9% and 71.7%, respectively.

### 3.2 The Pearson correlation analysis between MUVD, BMI, and VFA

**FIGURE 2:**
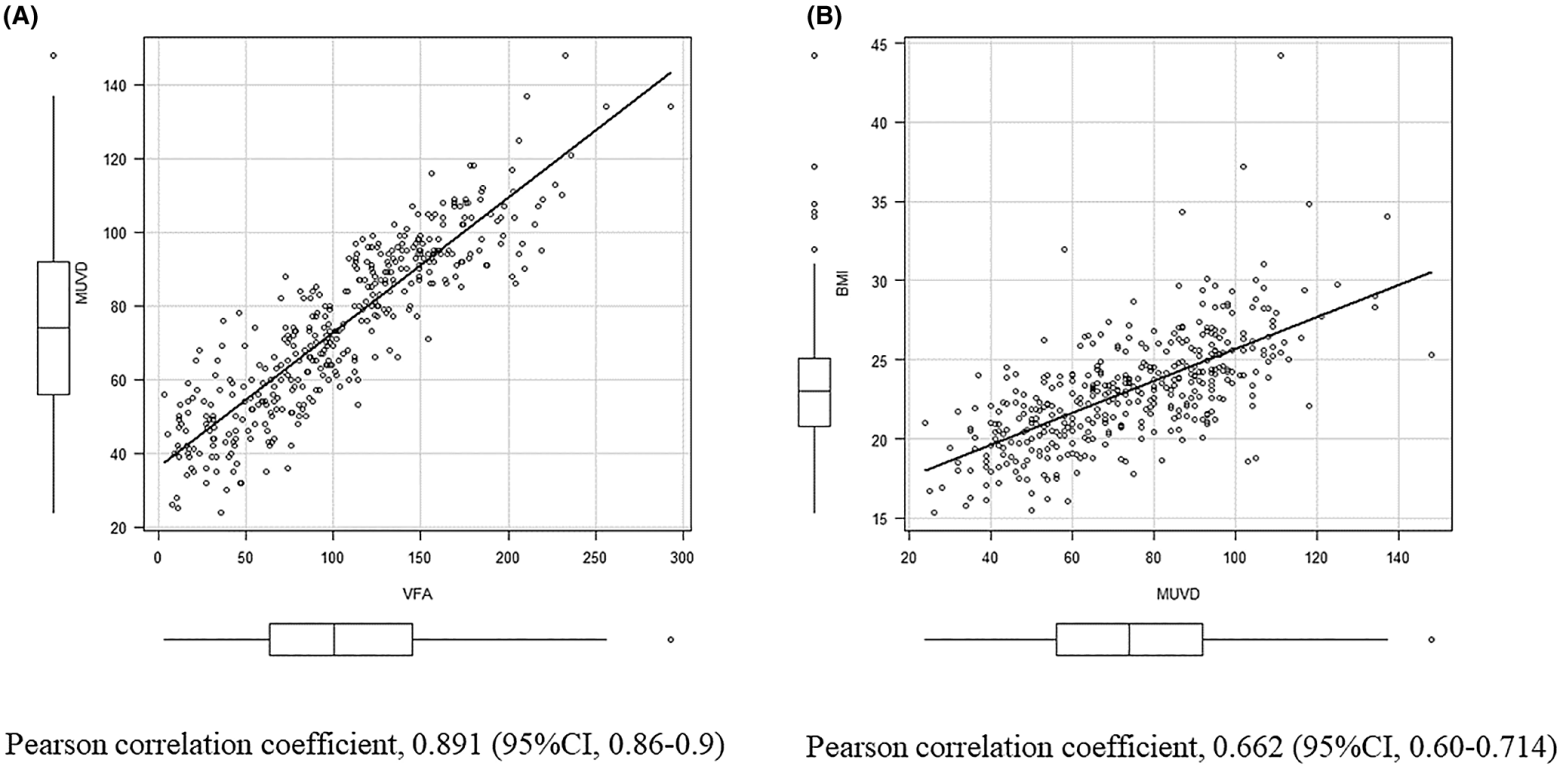
Pearson correlation analysis between MUVD and VFA (A) and between MUVD and BMI (B). Pearson correlation coefficient between MUVD and VFA and between MUVD and BMI are 0.891 and 0.662, respectively, indicating that MUVD has a very high correlation with VFA, whereas it has a high correlation with BMI.

When examining the correlation between MUVD, BMI, and VFA, the Pearson correlation coefficient between MUVD and VFA, and between MUVD and BMI were 0.879 and 0.53, respectively, indicating that MUVD has a very high correlation with VFA, while it has a high correlation with BMI (Figure 2).

### 3.3 Association between the MUVD value and patients background/short‐term outcomes in the whole cohort

Table 1 shows the comparison of patient characteristics and the short‐term postoperative results between the high and low MUVD group in all patients. The high MUVD group (*n* = 159) had significantly lower age (69.0 vs. 72.0, *p* = 0.039) and higher BMI (25.2 vs. 21.5, *p* < 0.01) and VFA (154.1 vs. 72.9 cm2, *p* < 0.01), and was more likely to be male (75.5% vs. 53.1%, *p* < 0.01) than the low MUVD group (*n* = 241). The high MUVD had more grade II and less grade I (*p* = 0.012) for the ASA‐PS criteria. Additionally, the high MUVD group had a higher rate of laparoscopic surgery (LG) and a lower rate of robotic surgery (RG) (*p* = 0.003). The patients were comparable in tumor stage, extent of gastric resection, and extent of lymph‐node dissection. Short‐term postoperative results showed that the high MUVD group had significantly longer operative time (376 vs. 362 min, *p* < 0.001) and greater blood loss (50.0 vs. 25.0 mL, *p* < 0.001). Postoperatively, there were significantly higher overall complication (25.2 vs. 12.0%, *p* = 0.001), severe complication (11.9 vs 4.1%, *p* = 0.005), overall IAICs (14.5 vs. 3.3%, *p* < 0.001), severe IAICs (8.8 vs. 2.1%, *p* = 0.003), pancreatic fistula (4.4 vs. 0.0%, *p* = 0.01), anastomotic leakage (6.3 vs. 1.2%, *p* = 0.008), and intra‐abdominal abscess (6.9 vs 2.1%, *p* = 0.019) in the high MUVD group. The median hospital stay was significantly longer in the high MUVD group than in the low MUVD group (12.0 vs. 11.0 days, *p* = 0.01).

**TABLE 1:** Baseline characteristics and surgical outcomes in patients who underwent minimally invasive gastrectomy (MIG) stratified by the MUVD value.

Evaluated variables	Total cases (*n* = 400)	Low MUVD (*n* = 241)	High MUVD (*n* = 159)	*p* value
Age^a^	71.0 (62.0–76.2)	72.0 (63.0–77.0)	69.0 (62.0–74.0)	0.039
Gender^b^				<0.001
Male	248 (62)	128 (53.1)	120 (75.5)	
Female	152 (38)	113 (46.9)	39 (24.5)	
ASA‐PS^b^				0.012
I	62 (15.5)	47 (19.6)	15 (9.4)	
II	288 (72.0)	162 (67.1)	126 (79.2)	
III	50 (12.5)	32 (13.3)	18 (11.3)	
BMI^a^	23.0 (20.7–25.0)	21.56 (19.7–23.4)	25.25 (23.4–26.7)	<0.001
VFA, cm^2^ ^a^	100.5 (63.9–145.2)	72.90 (41.5–95.7)	154.10 (131.7–175.7)	<0.001
MUVD, mm^a^	74.0 (56.0–92.0)	60.0 (49.0–71.0)	94.0 (90.0–102.0)	<0.001
cStage^b^				0.534
I	235 (58.8)	145 (60.2)	90 (56.6)	
II/III	165 (41.2)	96 (39.8)	69 (43.4)	
pT^b^				0.671
T1	237 (59.2)	148 (61.4)	89 (56.0)	
T2	45 (11.2)	26 (10.8)	19 (11.9)	
T3	73 (18.2)	40 (16.6)	33 (20.8)	
T4	45 (11.2)	27 (11.2)	18 (11.3)	
pN^b^				0.950
N0	263 (65.9)	158 (65.8)	105 (66.0)	
N1	73 (18.3)	45 (18.8)	28 (17.6)	
N2	29 (7.3)	18 (7.5)	11 (6.9)	
N3	34 (8.5)	19 (7.9)	15 (9.4)	
pStage^b^				0.289
I	237 (59.2)	147 (61.0)	90 (56.6)	
II	89 (22.2)	48 (19.9)	41 (25.8)	
III	73 (18.2)	46 (19.1)	27 (17.0)	
IV	1 (0.2)	0 (0.0)	1 (0.6)	
Type of surgical approach^b^				
Robotic	196 (49)	133 (55.2)	63 (39.6)	0.003
Laparoscopic	204 (51)	108 (44.8)	96 (60.4)	
Type of gastrectomy^b^				0.147
DG	338 (84.5)	203 (84.2)	135 (84.9)	
TG	49 (12.2)	27 (11.2)	22 (13.8)	
PG	13 (3.2)	11 (4.6)	2 (1.3)	
Extent of LN dissection^b^				0.121
D1/D1+	231 (57.8)	147 (61.0)	84 (52.8)	
D2	169 (42.2)	94 (39.0)	75 (47.2)	
Surgical time, min^a^	369.0 (318.0–436.0)	362.0 (307.0–420.0)	376.0 (338.0–453.5)	<0.001
Surgical blood loss, mL^a^	30.0 (11.5–70.0)	25.0 (10.0–60.0)	50.0 (27.50–85.0)	<0.001
The number of retrieved lymph nodes^a^	30.0 (22.0–39.0)	30.0 (23.0–41.0)	29.0 (21.0–35.0)	0.046
Clavien‐Dindo classification^b^				0.022
Grade 0	317 (79.2)	203 (84.2)	114 (71.7)	
Grade I	15 (3.8)	8 (3.3)	7 (4.4)	
Grade II	39 (9.8)	20 (8.3)	19 (11.9)	
Grade III	22 (5.5)	7 (2.9)	15 (9.4)	
Grade IV	4 (1.0)	2 (0.8)	2 (1.3)	
Grade V	3 (0.8)	1 (0.4)	2 (1.3)	
Overall complication^b^	69 (17.2)	29 (12.0)	40 (25.2)	0.001
Severe complication^b^	29 (7.2)	10 (4.1)	19 (11.9)	0.005
Overall IAICs^b^	31 (7.8)	8 (3.3)	23 (14.5)	<0.001
Severe IAICs^b^	19 (4.8)	5 (2.1)	14 (8.8)	0.003
Intraabdominal abscess^b^	16 (4.0)	5 (2.1)	11 (6.9)	0.019
Pancreatic fistula^b^	7 (1.8)	0 (0.0)	7 (4.4)	0.001
Anastomotic leakage^b^	13 (3.2)	3 (1.2)	10 (6.3)	0.008
Surgical site complication^b^	57 (14.2)	27 (11.2)	30 (18.9)	0.040
Systemic complication^b^	17 (4.3)	8 (3.3)	9 (5.8)	0.311
Reoperation^b^	9 (2.2)	5 (2.1)	4 (2.5)	0.745
Readmission^b^	11 (2.8)	6 (2.5)	5 (3.1)	0.760
Duration of postoperative stay, days^a^	11.0 (9.0–13.0)	11.0 (9.0–13.0)	12.00 (10.0–14.0)	0.001

Abbreviations: ASA‐PS, American Society of Anesthetists—Physical status; BMI, body mass index; DG, distal gastrectomy; IAICs, intraabdominal infectious complications; MUVD, Minimum umbilicus‐vertebra diameter; PG; proximal gastrectomy; TG, total gastrectomy; VFA, visceral fat area.

^a^ Data were expressed as median (interquartile range).

^b^ Data were expressed as number (%).

### 3.4 The multivariate analysis of risk factors for severe IAICs after MIG using the logistic regression model

Table 2 shows the results of the multivariate analysis of risk factors for severe IAICs after MIG. High MUVD (HR, 9.46, 95%CI,1.29–69.1, *p* = 0.026) and laparoscopic surgery (HR, 3.35, 95% CI, 1.040–10.80, *p* = 0.042) were independent risk factors for the occurrence of severe IAICs, whereas BMI and APD were not (*p* = 0.72 and *p* = 0.31, respectively).

**TABLE 2:** Multivariate analysis of risk factors for severe intra‐abdominal infectious complication following MIG for cancer.

Evaluated variables	Multivariate analysis		
	HR	95%CI *p* value	
Gender			
Male	1.73	0.509–5.890	0.379
Female	1.00		
Age			
≥75	2.37	0.845–6.640	0.101
<75	1.00		
BMI			
≥24	1.23	0.392–3.830	0.726
<24	1.00		
MUVD			
≥84 mm	9.46	1.290–69.100	0.026
<84 mm	1.00		
APD			
≥193 mm	0.34	0.043–2.760	0.317
<193 mm	1.00		
ASA‐PS			
3	1.79	0.493–6.470	0.377
1,2	1.00		
cStage			
II/III	1.42	0.509–3.940	0.505
I	1.00		
Type of gastrectomy			
TG and PG	1.94	0.542–6.460	0.281
DG	1.00		
Type of surgical approach			
Laparoscopy	3.35	1.040–10.80	0.042
Robot	1.00		

Abbreviations: APD, Anterior–Posterior Diameter; ASA‐PS, American Society of Anesthetists—Physical status; CI, confidence interval; DG, distal gastrectomy; HR, Hazard Ratio; MUVD, Minimum umbilicus‐vertebra diameter; PG, proximal gastrectomy; TG, total gastrectomy.

### 3.5 Association of surgical approaches (RG or LG) with baseline characteristics and surgical outcomes in patients with high MUVD using PS matching analysis

The backgrounds and surgical outcomes of patients who underwent RG or LG before and after PS matching in the high MUVD group are presented in Tables 3 and 4. Before PS matching, the RG group had more patients with grade III for the ASA‐PS criteria (*p* = 0.049). The RG group tended to have more males, more‐advanced oncological stage, and higher BMI than the LG group (*p* = 0.094, *p* = 0.098, and *p* = 0.073, respectively). Following PS matching, 51 patients in each RG and LG groups were selected. The groups were considered balanced for patient age, sex, ASA‐PS, oncological stage, and gastrectomy type. After PS matching, the median BMI and MUVD values in the RG and LG groups were similar (BMI, 25.1 vs. 24.8, *p* = 0.301; MUVD, 94 vs. 94, *p* = 0.611). Moreover, after PS matching, the RG group had a tendency for longer operation time; however, the RG group had significantly less surgical blood loss than the LG group (operation time, 407 vs. 371 min, *p* = 0.079; surgical blood loss, 40 vs. 60 mL, *p* = 0.005). Before PS matching, the patients in the RG group had significantly fewer severe IAICs (3.2% vs. 12.5%, *p* = 0.048). After PS matching, the severe IAIC rate tended to be lower in the RG group than that in the LG group (0% vs. 9.8%, *p* = 0.056).

**TABLE 3:** Characteristics of patients who underwent robotic gastrectomy (RG) and laparoscopic gastrectomy (LG) in the high MUVD group using propensity score matching analysis.

	Whole cohort			Matched cohort		
Characteristic	Robot (*n* = 63)	Laparoscopic (*n* = 96)	*p* value	Robot (*n* = 51)	Laparoscopic (*n* = 51)	*p* value
Age^a^	68.0 (63.5–73.0)	71.0 (61.5–74.0)	0.535	68.0 (63.5–73)	71.0 (61.5–74.0)	0.533
Gender^b^			0.094			1.00
Male	43 (68.3)	77 (80.2)		39 (76.5)	38 (74.5)	
Female	20 (31.7)	19 (19.7)		12 (23.5)	13 (25.5)	
ASA‐PS^b^			0.049			0.669
I	8 (12.7)	7 (7.3)		6 (11.8)	6 (11.8)	
II	44 (69.8)	82 (85.4)		40 (78.4)	39 (76.5)	
III	11 (17.5)	7 (7.3)		5 (9.8)	6 (11.8)	
BMI^a^	25.3 (18.5–37.2)	24.8 (18.80–44.22)	0.073	25.1 (23.5–27.5)	24.8 (22.6–26.2)	0.301
MUVD^a^	94.0 (84.0–148.0)	95.0 (84.0–137.0)	0.714	94.0 (91.0–103.0)	94.0 (89.0–102.0)	0.611
cStage^b^			0.25			0.842
I	32 (50.8)	58 (60.4)		30 (58.8)	28 (54.9)	
II/III	31 (49.2)	38 (39.6)		21 (41.2)	23 (45.1)	
pStage^b^			0.098			0.962
I	32 (50.8)	58 (60.4)		30 (58.8)	28 (54.9)	
II	15 (23.8)	26 (27.1)		12 (23.5)	14 (27.5)	
III	16 (25.4)	11 (11.5)		9 (17.6)	9 (17.6)	
IV	0	1 (1.0)		0	0	
Type of gastrectomy^b^			0.104			1.00
DG	55 (87.3)	80 (83.3)		45 (88.2)	46 (90.2)	
TG	6 (9.5)	16 (16.7)		6 (11.8)	5 (9.8)	
PG	2 (3.2)	0 (0.9)		0 (0.0)	0 (0.0)	
Extent of LN dissection^b^			1.00			0.692
D1/D1+	33 (52.4)	51 (52.8)		28 (54.9)	25 (49.0)	
D2	30 (47.6)	45 (47.2)		23 (45.1)	26 (51.0)	

Abbreviations: ASA‐PS, American Society of Anesthetists—Physical status; BMI, body mass index; DG, distal gastrectomy; LG, laparoscopic gastrectomy; MUVD, Minimum umbilicus‐vertebra diameter; PG, proximal gastrectomy; RG, robotic gastrectomy; TG, total gastrectomy; VFA, visceral fat area.

^a^ Data were expressed as median (interquartile range).

^b^ Data were expressed as number (%).

**TABLE 4:** Surgical outcomes in patients who underwent RG and LG in the high MUVD group using PS matching analysis.

Evaluated variables	Whole cohort			Matched cohort		
	Robot (*n* = 63)	Laparoscopic (*n* = 96)	*p* value	Robot (*n* = 51)	Laparoscopic (*n* = 51)	*p* value
Surgical time, mi^a^	409 (344–480)	371 (328–435)	0.019	407 (344–475)	371 (338–425)	0.079
Surgical blood loss, mL^a^	40.0 (20–70)	50.0 (30–102)	0.038	40 (20–75)	60 (42–125)	0.005
The mean number of retrieved lymph nodes^a^	30.0 (21–38)	29.0 (20–34)	0.714	30 (20–38)	29 (21–34)	0.535
Clavien–Dindo^b^			0.083			0.181
Grade 0	49 (77.8)	65 (67.7)		42 (82.4)	35 (73.2)	
Grade I	3 (4.8)	4 (4.2)		3 (5.9)	1 (2.0)	
Grade II	7 (11.1)	12 (12.5)		5 (9.8)	9 (17.6)	
Grade III	3 (3.2)	13 (13.5)		1 (2.0)	4 (7.8)	
Grade IV	2 (3.2)	0 (0.0)		0 (0.0)	0 (0.0)	
Grade V	0 (0.0)	2 (2.1)		0 (0.0)	2 (3.9)	
Overall complication^b^ ^,^ ^c^	13 (20.6)	27 (28.1)	0.351	8 (15.7)	14 (27.5)	0.228
Severe complication^b^ ^,^ ^d^	4 (6.3)	15 (15.6)	0.086	1 (2.0)	6 (11.8)	0.112
Overall IAICs^b^ ^,^ ^c^	7 (11.1)	16 (16.7)	0.366	3 (5.9)	8 (15.7)	0.200
Severe IAICs^b^ ^,^ ^d^	2 (3.2)	12 (12.5)	0.048	0 (0.0)	5 (9.8)	0.056
Pancreatic fistula^b^ ^,^ ^c^	1 (1.6)	6 (6.2)	0.245	1 (2.0)	3 (5.9)	0.617
Anastomotic leakage^b^ ^,^ ^c^	3 (4.8)	7 (7.3)	0.741	1 (2.0)	2 (3.9)	1.00
Intra‐abdominal abscess^b^ ^,^ ^c^	3 (4.8)	8 (8.3)	0.528	1 (2.0)	5 (9.8)	0.205
Reoperation^b^	1 (1.6)	3 (3.1)	1.00	0 (0.0)	2 (3.9)	0.495
Readmission^b^	2 (3.2)	3 (3.1)	1.00	2 (3.9)	1 (2.0)	1.00
Duration of postoperative stay, days^a^	11.0 (10.0–13.0)	12.0 (10.0–16.0)	0.106	11.0 (10.0–12.0)	12.0 (10.0–14.5)	0.037

Abbreviation: IAIC, intraabdominal infectious complication.

^a^ Data were expressed as median (interquartile range).

^b^ Data were expressed as number (%).

^c^ Clavien–Dindo classification Grade II or higher.

^d^ Clavien–Dindo classification Grade III or higher.

Regarding individual overall IAICs, no significant between‐group differences were noted (pancreatic fistula, 2.0% in the RG group vs. 5.9% in the LG group, *p* = 0.617; anastomotic leakage, 2.0% in the RG group vs. 3.9% in the LG group, *p* = 1.00; intra‐abdominal abscess, 2.0% in the RG group vs. 9.8% in the LG group, *p* = 0.205).

The RG group had a significantly shorter hospital stay following surgery than the LG group (11.0 vs. 12.0 days, *p* = 0.037).

## 4 DISCUSSION

In this study, we observed that the high MUVD group was associated with significantly more postoperative IAICs, particularly pancreatic fistula, anastomotic failure, and intra‐abdominal abscess, than the low MUVD group. Furthermore, multivariate analysis showed that the MUVD was an independent risk factor for severe IAIC occurrence. VFA has been previously reported to be a significant marker of IAICs.^20^, ^22^ In this study, the MUVD was noted to be highly correlated with VFA according to the Pearson correlation analysis. It has been reported that patients with a significant amount of visceral fat are more likely to have pancreatic leakage and intra‐abdominal abscess because the border between the pancreatic parenchyma and fat tissue is difficult to distinguish.^23^ Furthermore, as patients with visceral obesity bleed easily, recognizing the feasible layer for dissecting lymph nodes between the pancreas and fatty tissue is difficult, thereby increasing the possibility of causing pancreatic damage. It was speculated that the patients in the high MUVD group frequently have high VFA and are more likely to have severe IAICs. Measuring VFA requires specialized analysis software as opposed to MUVD measurement, which does not require such software and can be easily measured, making it useful for determining risk factors for IAICs before surgery in daily clinical practice. Conversely, although a high BMI is a marker of obesity that can be easily measured, it was not a significant risk factor for severe IAICs in the multivariate analysis. As BMI reflects the whole‐body muscle mass and fat mass compared with VFA and MUVD, it is assumed that BMI does not play a role in IAIC occurrence in gastric cancer surgery. It has been previously reported that VFA is a more significant marker than BMI for IAIC occurrence following gastric cancer surgery^22^, ^24^ which was consistent with this study.

Previous studies have reported that various measurements using axial CT sections affect short‐term surgical outcomes following gastrectomy. Ojima et al. reported that in male patients with gastric cancer who underwent LG, the APD was associated with the surgical time but not with the occurrence of postoperative complications.^12^ Lee et al. reported that female patients who experienced postoperative complications following open subtotal gastrectomy had higher APD values than those who did not.^13^ In this study, the AUC of the ROC curve for the MUVD tended to be higher than that of the APD (0.668 vs. 0.629, *p* = 0.092). Additionally, the multivariate analysis of risk factors for severe IAIC occurrence showed that the MUVD was an independent risk factor for severe IAICs, whereas APD was not. These findings suggested that the MUVD was a more reliable predictive marker for severe IAIC occurrence following MIG than BMI and APD. Yamamoto et al. reported that in cases of total gastrectomy and splenectomy, the distance from the epigastric skin to the root of the celiac artery at the upper abdominal body is involved in the occurrence of postoperative pancreatic fistula.^25^ They reported that when the pancreas is located deep, lymph node dissection around the pancreas becomes challenging, and pancreatic fluid leakage may occur more easily. Conversely, our study measured the distance from the umbilicus to the vertebral body in a CT slice at the umbilical level, which is presumed to reflect the amount of visceral fat rather than the pancreatic depth. To our knowledge, no previous studies have reported the relationship between the MUVD and severe IAICs. The multivariate analysis of severe IAICs risks showed that in addition to the MUVD, robotic surgery was an independent significant factor for preventing severe IAIC occurrence. We previously reported that RG reduced severe IAICs following MIG more frequently compared to LG.^17^ Therefore, we also examined the impact of RG on severe IAICs in the high MUVD group using PS matching analysis. We observed that RG had a significantly lower severe IAIC rate before PS matching and a tendency for fewer severe IAICs after PS matching in the high MUVD group than that in the LG group. Recently, Hikage et al.^26^ reported that the incidence rates of overall IAICs in patients with high VFA were lower in the RG group than those in the LG group. Robotic systems have several advantages, including an articulated endo‐wrist, tremor filtering, and a superior surgical view. These advantages enable us to perform safe and meticulous lymph node dissection around the pancreas, which may result in less severe IAICs in patients with visceral obesity. Therefore, from the abovementioned results, a treatment strategy of introducing RG for the high MUVD group is considered useful from the perspective of preventing severe IAICs.

### 4.1 Limitations

This report is a single‐center retrospective review. In addition, it has been reported that postoperative complications are correlated with the skill and experience of the surgeon, thus results may vary depending on these factors. In this study, both robotic and laparoscopic surgery were performed by well‐experienced surgeons who are certified by the Japanese Society of Endoscopic Surgery. Because patients who do not qualify were excluded from this study, it is assumed that bias in technique and experience among surgeons was minimized. Additionally, since this study targeted only Japanese patients, this MUVD cutoff value may be suitable only for Japanese patients. It may be necessary to consider them individually in Europe, the United States, and other Asian countries. In addition, under special circumstances such as ascites retention, intestinal obstruction, or multiple renal cysts, MUVD may increase even though visceral fat is low. Such cases need to be excluded in this study.

## 5 CONCLUSION

The MUVD was a novel and easy‐measuring predictor of severe IAICs following MIG. Robotic surgery should be considered first in patients with gastric cancer having an MUVD value of 84 mm or higher from the perspective of severe IAIC occurrence.

## AUTHOR CONTRIBUTIONS

Study Design: NK. Data collection: NK, KS, TH, JN, YI, TN, SS, and TI. Statistical analysis and interpretation of results: NK. Drafting of the manuscript: NK. Supervision: KM and YN.

## FUNDING INFORMATION

This study did not receive support from any organization.

## CONFLICT OF INTEREST STATEMENT

The authors declare no conflicts of interest for this article.

## ETHICS STATEMENT

Approval of the research protocol: This study was conducted in accordance with the ethical principles of the Declaration of Helsinki. This was a retrospective study approved by the review board of Osaka City General Hospital.

Informed consent: Patients provided written informed consent.

Registry and the Registration No. of the study/trial: N/A.

Animal Studies: N/A.
